# Optimized Isolation of Extracellular Vesicles From Various Organic Sources Using Aqueous Two-Phase System

**DOI:** 10.1038/s41598-019-55477-0

**Published:** 2019-12-16

**Authors:** Oğuz Kaan Kırbaş, Batuhan Turhan Bozkurt, Ayla Burçin Asutay, Beyza Mat, Bihter Ozdemir, Dilek Öztürkoğlu, Hülya Ölmez, Zeynep İşlek, Fikrettin Şahin, Pakize Neslihan Taşlı

**Affiliations:** 1 Yeditepe University, Faculty of Engineering and Architecture, Department of Genetics and Bioengineering, Kayisdagi St., 34755 Istanbul, Turkey; 2 Tubitak Marmara Research Center, Baris Dist, 41470 Kocaeli, Turkey

**Keywords:** Nanobiotechnology, Exocytosis, Multivesicular bodies, Secretion

## Abstract

From biomarkers to drug carriers, Extracellular Vesicles (EVs) are being used successfully in numerous applications. However, while the subject has been steadily rising in popularity, current methods of isolating EVs are lagging behind, incapable of isolating EVs at a high enough quantity or quality while also requiring expensive, specialized equipment. The “isolation problem” is one of the major obstacles in the field of EV research - and even more so for their potential, widespread use for clinical diagnosis and therapeutic applications. Aqueous Two-Phase Systems (ATPS) has been reported previously as a promising method for isolating EVs quickly and efficiently, and with little contaminants - however, this method has not seen widespread use. In this study, an ATPS-based isolation protocol is used to isolate small EVs from plant, cell culture, and parasite culture sources. Isolated EVs were characterized in surface markers, size, and morphological manner. Additionally, the capacity of ATPS-based EV isolation in removing different contaminants was shown by measuring protein, fatty acid, acid, and phenol red levels of the final isolate. In conclusion, we have shown that EVs originating from different biological sources can be isolated successfully in a cost-effective and user-friendly manner with the use of aqueous two-phase systems.

## Introduction

Extracellular vesicles (EVs) are lipid-bilayered nanoparticles released from all cell types^1^. They act as cellular messengers, with specific cargo and characteristics based on the cells that they derive from. Characteristic properties of EVs are determined by the physiological state of the cell producing it, and these EVs can in turn alter the function and physiology of other cells^2^. EVs has been categorized into many different subdivisions based on their physical characteristics, surface markers and biogenesis.

Due to their role as natural messengers, EVs have been the focus of many studies investigating their potential as disease biomarkers and carriers for drugs and nucleic acid complexes, such as CRISPR-Cas9^3^. EVs exhibit several advantages over synthetic drug-carrying vesicles. Compared to synthetic carriers, such as liposomes, EVs such as exosomes can last longer in the circulation by avoiding phagocytosis by monocytes^4,5^. Additionally, unlike synthetic drug carriers such as liposomes, which may cause some toxicity^6,7^, EVs have little to no adverse effects in the body^8^. EVs also more readily avoid endosomal pathways and lysosomal degradation compared to synthetic carriers, allowing better delivery of fragile cargos, such as siRNA or CRISPR complexes^9^.

EVs show specificity towards the type of cells that they originate from via cell-specific membrane-bound proteins that they carry, allowing a degree of cell homing for the delivery of a therapeutic *in vivo*. EVs from different cells can be engineered to show specificity towards other cells, such as cancer cells for drug delivery^10^. EVs are also capable of carrying drugs through the blood-brain-barrier - even ones that would not pass the brain on their own^11^. Cargo that EVs carry -proteins and nucleic acids, especially miRNAs- also make them a valuable source of diagnostic markers^12–15^.

Many of these studies have focused on using circulating EVs to diagnose different types of cancer, especially for types of cancer that are harder to diagnose. While these applications of EVs are shown to have great potential for a new generation of diagnosis and treatment options, many of these currently struggle to enter practice. Greatest challenge these applications face is the unavailability of an EV isolation protocol that can isolate EVs efficiently and with a high purity, and using devices that are currently available at hospitals and similar institutions. Additionally, many of the isolation methods -including the golden standard, ultracentrifugation- damage the structure of the EVs due to the forces involved in isolating these tiny particles. EV isolation methods of ultracentrifugation, ultrafiltration and immuno-affinity columns are capable of isolating highly pure EVs, at the cost of excessively long isolation times that result with a small number of EVs at the end. Polymeric precipitation of EVs, which allows their isolation at normal centrifugation speeds, results with contaminating proteins and other extracellular vesicles^16^. Additionally, these isolation methods are incapable of producing EVs at a larger scale, which would reduce the production costs of EV-based therapeutics. New and upcoming methods, such as using magnetic nanoparticles^17^, or tangential flow filtration^18^ attempt to provide a solution to the EV isolation problem. Different methods of EV isolation are compared under Table 1, and the subject has been reviewed deeply by Konoshenko *et al*.^19^.

**Table 1:** Comparison of commonly used EV isolation methods.

Methods	Advantages	Disadvantages
Ultracentrifugation based methods	Generally pure samples with low/no operational costs	Cost of equipment, long isolation times, complexity - many variables to consider
Differential Ultracentrifugation^32–35^	High working volume	Damage and deformation of EVs, protein contamination, low reproducibility.
Density Gradient Ultracentrifugation^36^	Pure isolates	Low efficiency, complexity, exosomal aggregation, viral particles migrate to the same density gradient with the EVs.
Density Cushion Ultracentrifugation^37^	Pure isolates, gentle	Low efficiency, small sample volume, complexity.
Size based methods	Simple process, can handle continuous processing	Filter clogging, shear and deformation of EVs, low process rate.
Ultrafiltration^38,39^	Simple process, can handle continuous processing	Filter clogging, shear and deformation of EVs, low process rate.
Tangential flow filtration^18,40^	Pure, scalable, reproducible, gentle on the integrity of EVs	Complex setup
Size exclusion chromatography^41,42^	Gentle isolation	Long running times
Affinity based methods	Pure isolates and efficiency	High running costs
Antibody coated beads^43^	Pure isolates	Damage to surface proteins, cost
Magnetic beads with Tim4^17^	Pure isolates, gentle on the EVs	Consumable cost, does not work at presence of organic acids
Aqueous two phase isolation, PEG + Dextran^20,21^	Cheap, high efficiency and purity, scalability, removes non-protein contaminants.	Presence of dextran at the final sample
Precipitation based methods	Requires no need for specialized equipment, fast isolation	Very high degrees of contaminants, presence of polymers
Polymeric precipitation^44^	Simple process, no need for specialized equipment	High protein contaminants, presence of polymer, high consumable cost
Antibody mediated precipitation^45^	Highly pure samples with relatively high efficiency	High cost of consumables.
Sodium acetate precipitation^46^	Low cost	Protein contaminants

Aqueous two-phase systems (ATPS) are formed by mixing two polymers, or polymers with salt solutions used to separate and isolate a variety of biomolecules. Shin *et al*. have published a method for isolating EVs using an ATPS system formed by mixing polyethylene glycol (PEG) and dextran (DEX)^20^, and later, Kim *et al*. added further steps to remove contaminating proteins from the isolated EVs^21^. ATPS-based isolation of EVs has a distinct advantage over previous isolation methods, being able to isolate EVs at high efficiency and purity while requiring less time and no specialized equipment. The primary disadvantage of using ATPS to isolate EVs is the presence of dextran in the final suspension of EVs. Dextran interferes with common analytical techniques such as Western Blotting or RNA isolation, affects the viability of cells *in vitro* and increases the viscosity of the samples, which results in a more heterogeneous distribution of EVs.

In this study, we present a modification of the previously described ATPS EV isolation method that optimizes the workflow of EV production. We have isolated and characterised EVs from mammalian and parasite culture media, as well as serum samples. We have also isolated EV-like plant nanoparticles (plant NPs). Washing steps of the ATPS-EV isolation method is capable of removing phytochemicals and other contaminants from the final isolate of EVs which may interfere with certain assays and studies.

## Results

### Characterization of the ATPS system

**Figure 1:**
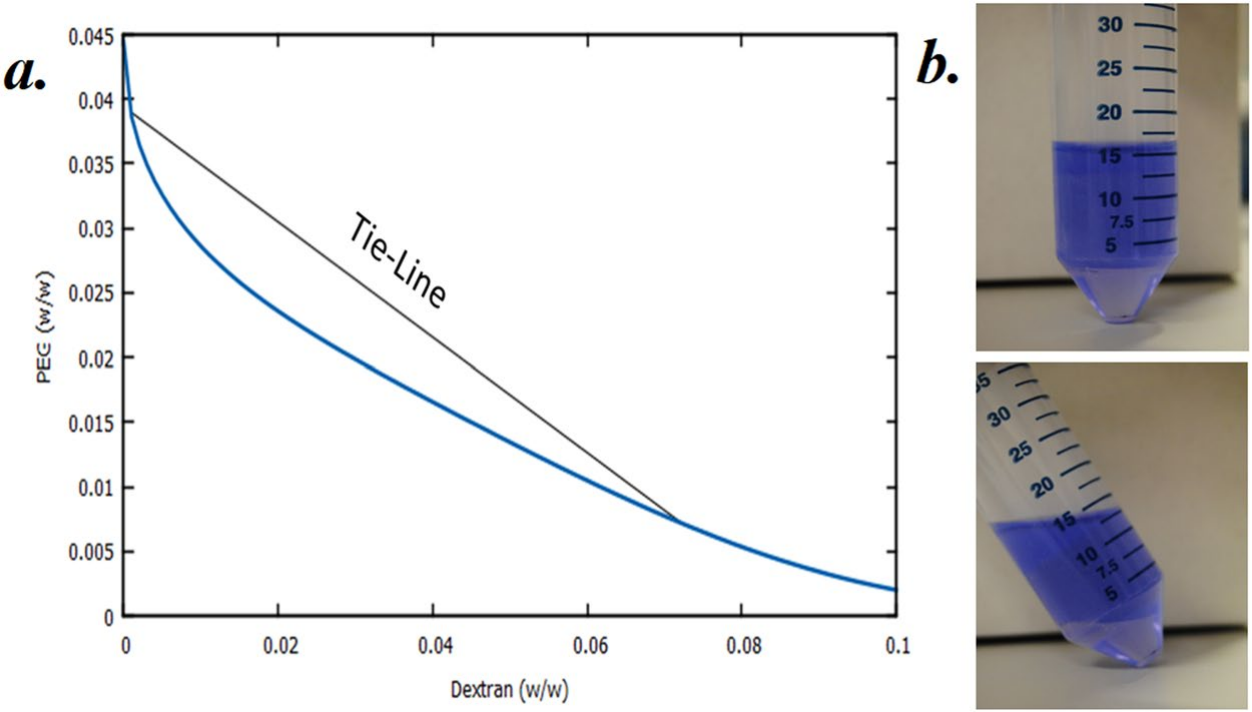
Binodal curve of PEG/Dextran ATPS, and tie line of the system.

Physical properties of an ATPS system depends on the composition of its elements. Binodal curves are used to identify at which concentrations components of an ATPS separate into two distinct volumes. If concentrations of the components that form the ATPS are at the biphasic region (ie. above the binodal) of that particular system, the components segregate into two distinct phases. Different points at the biphasic curve form ATPS with different physical properties, such as phase compositions, volumes of each phase, and the interfacial tension between the phases. Points at the biphasic region that are on the same tie-line results in systems with same composition of phase-forming components at the separated phases, resulting in systems with same interfacial tensions (Fig. 1a)^22^. Presence of a well-defined interfacial layer was visually confirmed by adding Coomassie Brilliant Blue R-250, which selectively migrates to the top phase of the ATPS (Fig. 1b). Composition of the equilibrated phases were determined via measuring the density and volumes of the phases (Table 2).

**Table 2:** Physical properties and compositions of the equilibrated phases.

	Top Phase	Bottom Phase
Volume (ml)	37.3	2.7
Density (g/ml)	1.00750	1.02550
%PEG (w/w)	3.90	0.72
% Dextran (w/w)	0.09	7.22

### Characterization of plant, parasite, serum and cell culture EVs isolated with ATPS-EV isolation method

In order to show that the ATPS method is capable of isolating EVs from different biological samples, protein markers and morphological features of isolated EVs were characterized in accordance with the MISEV2018 guidelines^1^.

**Figure 2:**
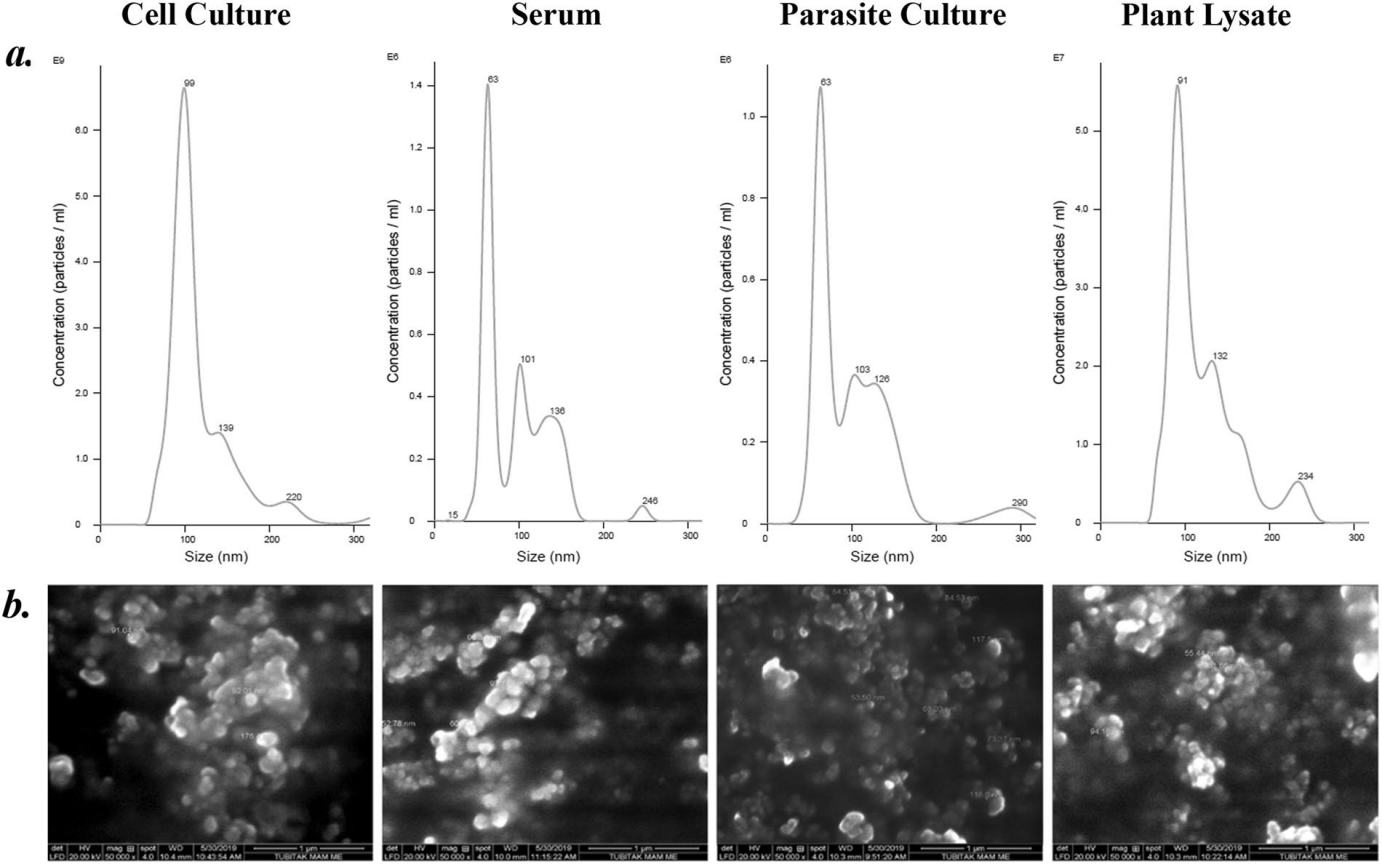
Physical characterisation of isolated cell culture, serum and parasite EVs and plant EV-like NPs (**a**) NTA size distributions of the samples. (**b**) SEM imaging of the isolated EVs. Uncut size distributions and additional SEM images are available in the supplementary figure.

Physical characterization of the EVs were done with the complementing methods of Nanoparticle Tracking Analysis (NTA), Dynamic Light Scattering (DLS) and Environmental Scanning Electron Microscopy (ESEM) to determine their size and morphology. EVs from all four origins were comparable to one another with respect to their physical characteristics (Fig. 2). NTA results showed a characteristic distribution of peaks for EVs - a major peak between 30–100 nm in diameter, followed by a minor peak between 100–200 nm in diameter (Fig. 2a). Uncut graphs of NTA size distributions can be seen in Supplementary Fig. 1. These results were confirmed via SEM imaging, where they measured between 60–200 nm (Fig. 2b) in diameter, and showed a homogeneous distribution of similarly sized particles. Wide-range SEM shots of samples was provided in Supplementary Fig. 2.

**Figure 3:**
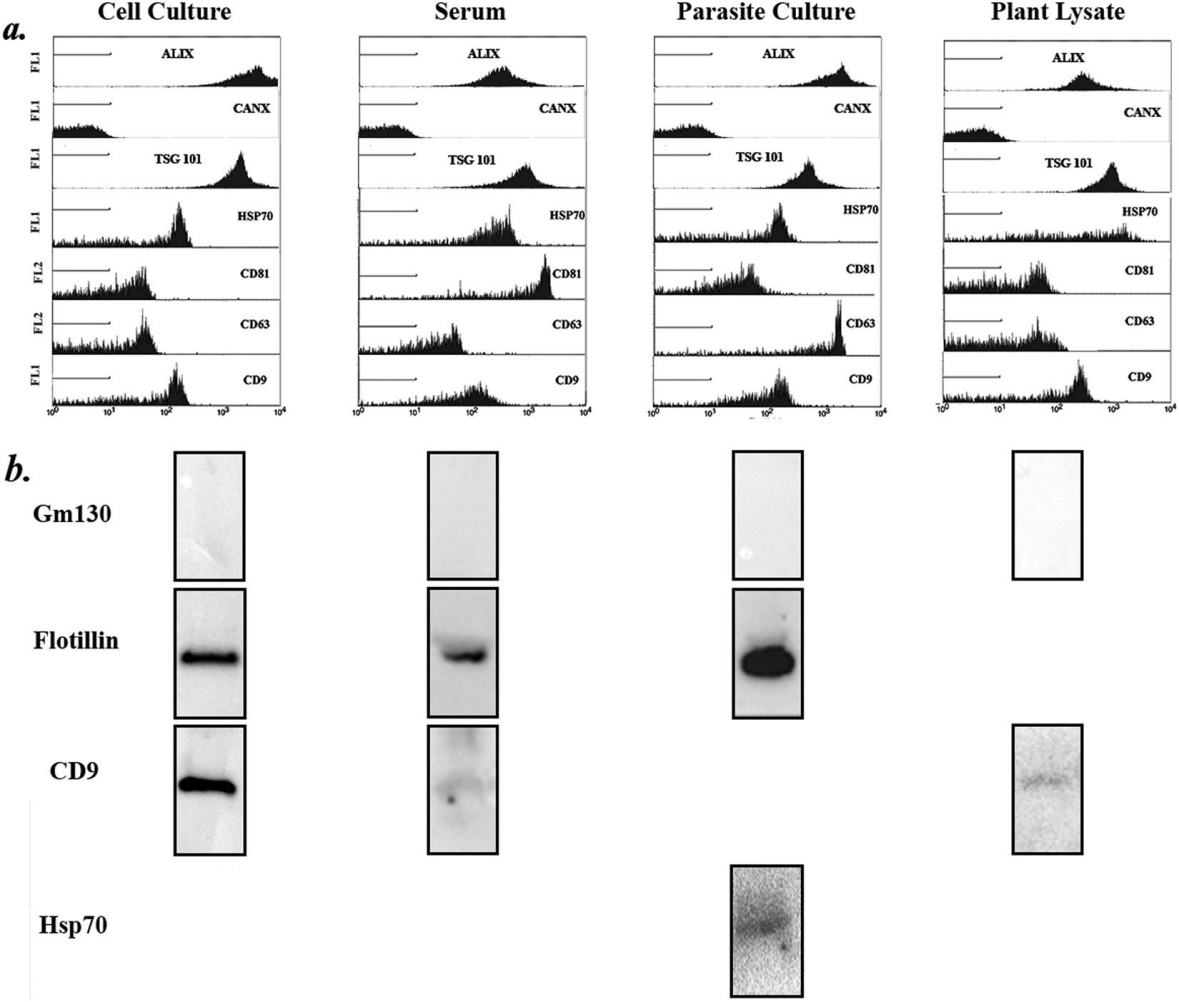
Biomarker characterisation of isolated cell culture, serum and parasite EVs and plant EV-like NPs (**a**) Flow Cytometry of common EV surface markers (ALIX, CANX, TSG101, HSP 70, CD 81, CD 63 and CD 9). (**b**) Cropped images of Western Blots of select EV markers (GM130, Flotillin, CD9 and HSP70. All markers were run on the same lane owing to their different molecular weights. Full length gel images are included in Supplementary Fig. 3.

Biological characterization of isolated EVs were done via the flow Cytometry and Western Blotting of common EV markers - CD9, CD63, CD81, HSP70 TSG101, Alix and Flotillin. Calnexin (CANX) or GM130 were used as Flow Cytometry and Western Blotting negative markers respectively. EVs of cell culture and serum origin were shown to be positive for all markers tested, except the negative marker CANX, in both Flow Cytometry (Fig. 3a) and Western Blot analysis (Fig. 3b). Parasite EVs and plant NPs were only positive for all markers except the negative markers in Flow Cytometry, and were positive for HSP70 or CD9 respectively, and was negative for GM130 in Western Blot analysis. Whole-gel images of Western Blots were provided in Supplementary Fig. 3.

### Removal of protein contaminants with the ATPS-EV isolation method

EV isolation protocols must include steps that prevent the co-isolation of soluble protein. Proper removal of soluble proteins from the EV isolate prevents any interference to experiments due to these proteins, and allows accurate EV quantification through total protein quantification.

**Figure 4:**
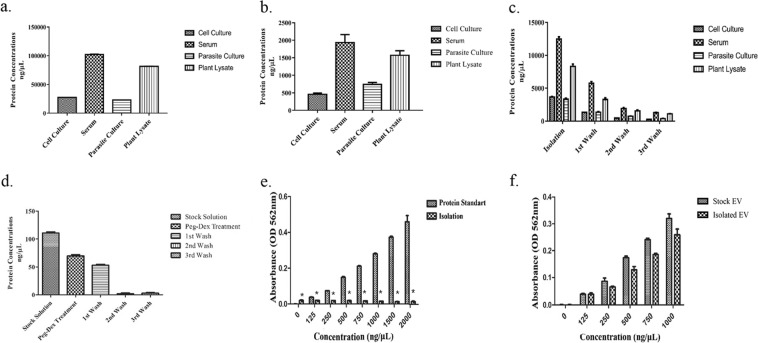
Isolation efficiency and capacity to remove protein contaminants of the ATPS-based EV isolation protocol. (**a**) Initial protein concentrations of EV containing biofluids. (**b**) Final concentrations of EVs isolated from different biofluids. (**c**) Protein concentrations of samples at different steps of the isolation process. (**d**) BCA measurement of 100 *μ*g/mL BSA samples at various steps of isolation. (**e**) BCA measurement of various concentrations of BSA after two steps of washing. Protein standards were compared to their pre-isolation counterpart for significance (**f**) Pre-and-post isolation concentrations of EV samples with a range of starting EV concentrations.

Total protein concentrations of each EV source that were used (Fig. 4a), and the concentrations of EVs that were isolated from equal volumes of these different sources were measured (Fig. 4b). Serum and plant lysate samples had higher protein concentrations in both their initial concentrations and EV isolates than culture-derived sources. Protein levels were measured throughout different steps of the isolation process for all four samples (Fig. 4c) to determine the optimal number of washing steps for each sample.

In order to validate the protein removal capabilities of the method, a standard solution of 100 *μ*g/mL of BSA was run through the isolation process. Protein concentrations was dropped 62% after the first wash, and was reduced below the detection level after the second washing step (Fig. 4d). To show that the protein removal capabilities of the system does not get over-saturated at higher concentrations of contaminating proteins, a set of 8 different protein with various concentrations of BSA was run through the isolation process with three washing steps. Differences between the final protein concentrations post-third wash between different starting concentrations of proteins were insignificant (Fig. 4e). Finally, in order to determine the amount of EVs lost through an EV sample was prepared with a three-wash isolation was prepared into 5 EV samples with known concentrations. On average, there was an 8% loss to EV concentrations post-third wash (Fig. 4f).

### Removal of non-protein contaminants with the ATPS-EV isolation method

**Figure 5:**
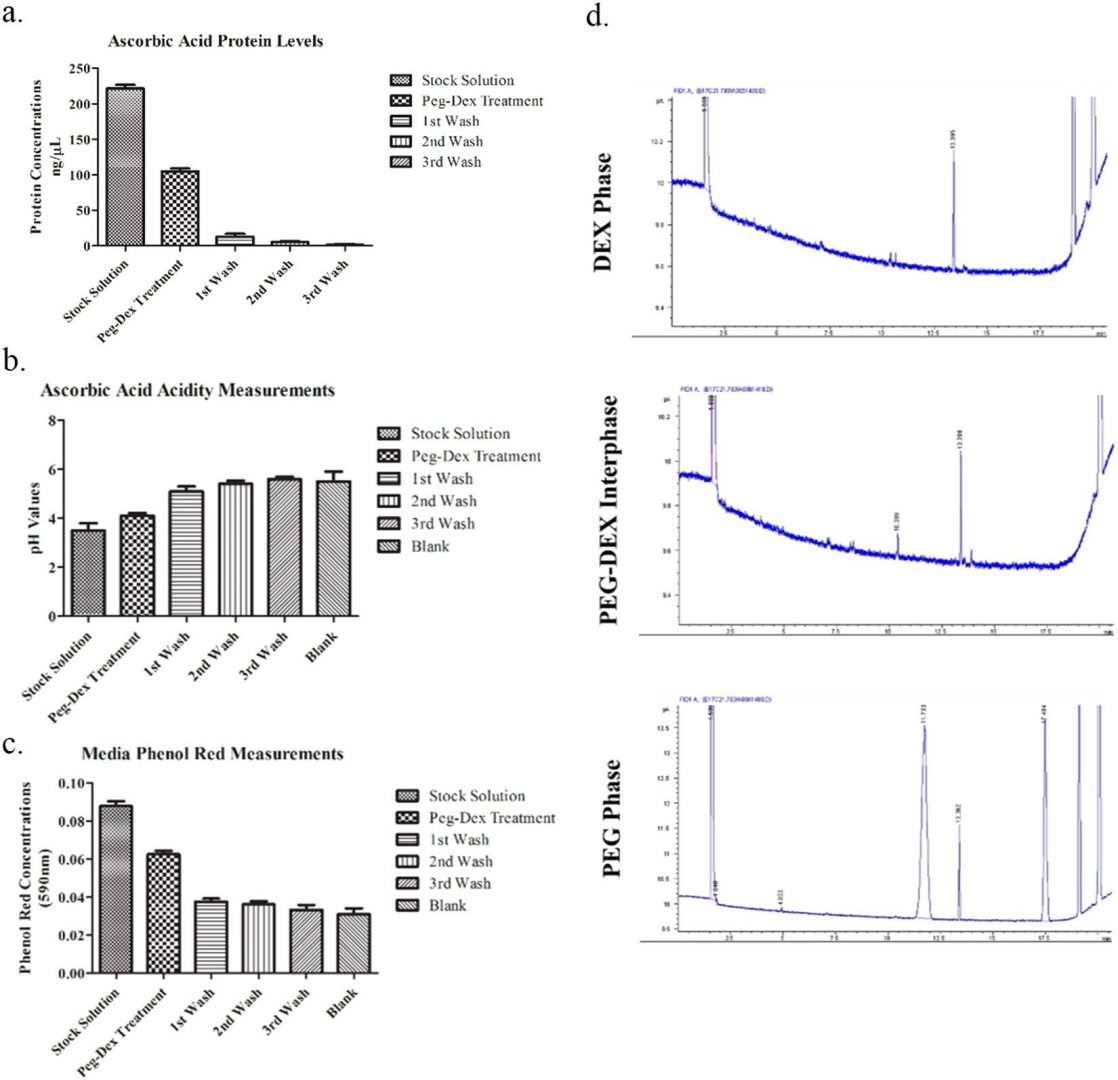
Capacity of the ATPS-based EV isolation protocol in removing non-protein contaminants. (**b**) BCA measurement of ascorbic acid, which cause false positives in BCA, at different steps of isolation (**b**) Changes in acidity after removal of ascorbic acid. (**c**) Removal of phenol red from culture media samples, measured via absorbance at 590 nm. (**d**) FAME anaylsis by gas chromatography showing the removal of lipid contaminants.

Proteins are only one of possible contaminants of EV isolation. Different downstream applications may also be incompatible with different non-protein contaminants. New EV sources such as edible plants may contain compounds such as ascorbic acid that interfere with common assays in EV studies, such as EV quantification through BCA assay. False-positive protein concentration measurements due to a simulated ascorbic acid contamination, was alleviated with the washing steps of the ATPS-EV isolation (Fig. 5a). Reduction of the ascorbic acid levels were also confirmed via measuring the pH of the isolates at different steps of isolation (Fig. 5b). Samples rapidly reached neutral pH after the first washing step and onwards.

Reduction in phenol red levels at different steps of EV isolation was measured via spectrophotometric analysis. Absorbance difference caused by phenol red became insignificant after first washing step (Fig. 5c).

Finally, contaminating lipids may have functional effects similar to contaminating proteins, and may cause false results. *α*-linoleic acid was used to simulate a possible lipid contaminant of EV isolations. FAME analysis of the EV containing dextran phase, and the upper PEG phase confirmed that lipid contaminants could be removed via ATPS-based EV isolation method (Fig. 5d).

## Discussion

Even though researchers interest on EVs -especially exosomes- has greatly increased during recent years, the current methods of EV isolation are lagging behind the demands of the field. In particular, studies involving non mammalian EVs has attracted interest - plants, parasites^23^, fungi^24^ and even prokaryotes^25^ have been reported to secrete EVs. Plant exosome-like nanoparticles are reported to affect mammalian cells^26,27^, and have anticancer properties^28^. Current methods of EV isolation were developed mostly with mammalian cell culture and body fluids in mind. Even with the development of advanced methods of EV isolation, such as the use of of Tim4 proteins affinity towards phosphatidylserine of exosomal membranes for EV isolation^17^, may struggle adapting to different samples such as plants and blood samples, as due to the presence of organic acids and EDTA present, in plants and some blood collection tubes respectively. Calcium chelators^29^, prevent the interaction between Tim4 and EVs, making such an isolation impossible. Developing new methods of EV isolation that are efficient, easy-to-use, and adaptable to the challenges of the new EV sources are vital for advances in the EV field. Aqueous two phase systems were reported as a way of isolating EVs at a high efficiency^20^, and purity^21^.

In this study, we have successfully isolated EVs from plant lysate, blood serum, parasite culture and mammalian cell culture samples using an ATPS-based isolation method. We have also characterized the physical properties of the ATPS used. We have found that in addition to removing contaminating proteins^21^, ATPS-based isolation was capable of purifying EVs from different contaminants such as fatty acids, phenol red or plant phytochemicals, which could affect downstream assays such as BCA. By using mock-samples prepared with particular contaminants, we determined the optimal number of washes required for the removal of these contaminants for such samples was two. Different samples and applications may require additional washes.

Partitioning properties of aqueous two-phase systems along the same tie-line are identical to one another. However, while the composition of the ATPS components along the tie-line does not change, volumes of the separated phases change. By choosing a system on the tie-line that contains higher concentrations of PEG, one can manipulate the volume of the dextran-rich bottom phase to be as low as possible, allowing a more concentrated EV isolate as a result. Compositions of PEG and dextran on each phase are calculated and provided in Table 2.

Isolated EVs were characterized in accordance to the MISEV2018^1^ standards. While particles isolated from the plant lysate are not purely formed out of extracellular vesicles -due to release of intracellular vesicles during plant lysis- these nanoparticles exhibit EV-like characteristics; such as size distribution, and expression of EV biomarker proteins.

Physical characterization of all samples showed an enrichment towards <200 nm. Characterization of EV biomarkers was done via complementing methods of Flow Cytometry and Western Blotting. Flow Cytometry results were compliant with literature on EV^1^.

Expression of common EV biomarkers on plant EV-like NPs was shown for the first time according to our literature search. The fact that human-reactive antibodies were capable of binding with these is a proof towards the evolutionary conservation of EV secretion across species. Indeed, a homolog of CD63, *TET8* was used to label EVs from *Arabidopsis*^30^.

Success of an EV isolation protocol greatly depends on its ability to produce highly pure products. Effects attributed to a particular EV may be due to the co-isolated proteins that come with said EV. Additionally, total protein quantification is a commonly used method for EV quantification, and presence of such proteins may cause overestimation of EV concentrations by users. By running the isolation process with samples containing various concentrations of BSA, we have determined that a two-step washing process is capable of removing all contaminating proteins, even at higher protein concentrations (Fig. 4e). In order to determine the loss of EVs due to the washing process, we have also prepared a range of samples with known concentrations of EVs, and ran them through the EV isolation process. By comparing their protein concentrations before and after isolation, we have determined that an average of 8% of EVs are lost due during washing, and the starting EV concentrations does not affect this loss.

In addition to proteins, non-protein contaminants can affect studies involving EVs. While plants are new and exciting EV source, isolation of their EVs through conventional means is a great challenge. Papers that work with plant lysates has to handle large particles and fragments of the lysate, which require additional centrifugation on top of the standart sequential centrifugation of ultracentrifugation based EV isolation methods. Additionally, many phytochemicals of plant EVs may end up co-isolated with the EVs, and may cause issues with downstream applications and assays. For example, plant acids may cause false large false positives with BCA assay, which may cause an overestimation of EV concentrations (Fig. 5a). Ascorbic acid was used as a representative plant acid contaminant to deduce if the ATPS-based EV isolation protocol is capable of removing such contaminants. A two-step wash was capable of reducing ascorbic acid concentrations below detectable level (Fig. 5b). Finally, common cell culture media pH indicator, phenol red, may cause issues with colorimetric measurements. Phenol red levels were statistically equivalent after the first wash.

Presence of dextran, present within the final phase of EV isolation is the major drawback of the system. Dextran increases the viscosity of the sample leading to a more heterogeneous distribution and causes distruptions when ran in gel electrophoresis studies, is insoluble in monohydric alcohols and ketones, which prevents their use in methods such as RNA isolation. Additionally, when using EVs isolated with this method, effects of dextran itself must be considered for the study. Negative control samples may be given “dextran blanks”, prepared by going through the isolation protocol with PBS or fresh culture media.

In conclusion, we have shown that the EVs originated from different biological sources can be isolated successfully in a cost-effective and user-friendly manner by aqueous two-phase systems. Contrary to traditional EV isolation methods, EVs can be purified from various biological contaminants like fatty acids, organic acids and most of the bioactive compounds as well as non-exosomal proteins. Potential adverse effects of dextran polymer that is present in EV-rich phase ought to be investigated in future.

## Methods

### Cell culture

HaCaT cell line was used as a mammalian cell source. HaCaT cells were cultured in complete growth medium which is composed of Dulbecco’s modified Eagle’s medium (DMEM) supplemented with 10% (v/v) Fetal Bovine Serum (FBS) and 1% (v/v) Penicillin-Streptomycin-Amphotericin (PSA) (10,000 units/mL penicillin, 10,000 *μ*g/mL streptomycin, 25 *μ*g/mL amphotericin B; Invitrogen, Gibco, UK). After reaching 80’“90% confluency, the cells were transferred to a T-150 flask (Zelkultur Flaschen, Switzerland) at a density of 500,000 cells/flask and were maintained at 37 °C and 5% CO_2_ in a humidified incubator. One day after seeding HaCaT cells in flasks, complete growth medium was replaced by the EV collection medium that consisted of DMEM, 10% (v/v) Exosome Depleted FBS (Gibco) and 1% (v/v) PSA. Culture media was collected one day after the media change. Cell confluency was at 60 to 70% at the time of media collection, and no dead cells were observed at the time of media collection. Culture media was stored at −20 °C before EV isolation.

### Parasite culture

*Leishmania infantum* promastigotes were kindly provided by the Institute for Molecular And Cell Biology (IBMC), University of Porto (Portugal). *Leishmania infantum* promastigotes were grown at 25°C in RPMI 1640 Glutamax (Gibco), complemented with 10% (v/v) Exosome Depleted FBS (Gibco), 50 U mL-1 penicillin, 50 *μ*g/mL streptomycin, and 20 mM HEPES sodium salt pH 7.4 (Sigma). After 7–10 days stationary phase cultures Parasites were expanded until they differentiated into infectious metacyclic promastigotes^31^. Parasite culture media was collected at the end of the differentiation, and stored at −20 °C before EV isolation. No dead cells were observed at the time of media collection.

### Preparation of the ATPS isolation solution

ATPS-EV isolation solution was prepared by dissolving Poly(ethylene glycol) (PEG) (Sigma, 81310) and Dextran (DEX) from Leuconostoc spp. (Sigma, 31392) in distilled water at a 7,7:3,3 (w/w) ratio as described in Table 3.

**Table 3:** Preparation of ATPS isolation solutions.

Chemical Name	Molecular Weight (MW)	Linear Molecular Formula	Solvent	Concentration (%)
Poly ethylene glycol	25,000–45,000	H(OCH2CH)_n_OH	dH_2_O	3,35 w/v
Dextran	450,000–650,000	(C6H10O5)_n_	dH_2_O	1,65 w/v

### Isolation of EVs using ATPS-EV isolation method

**Figure 6:**
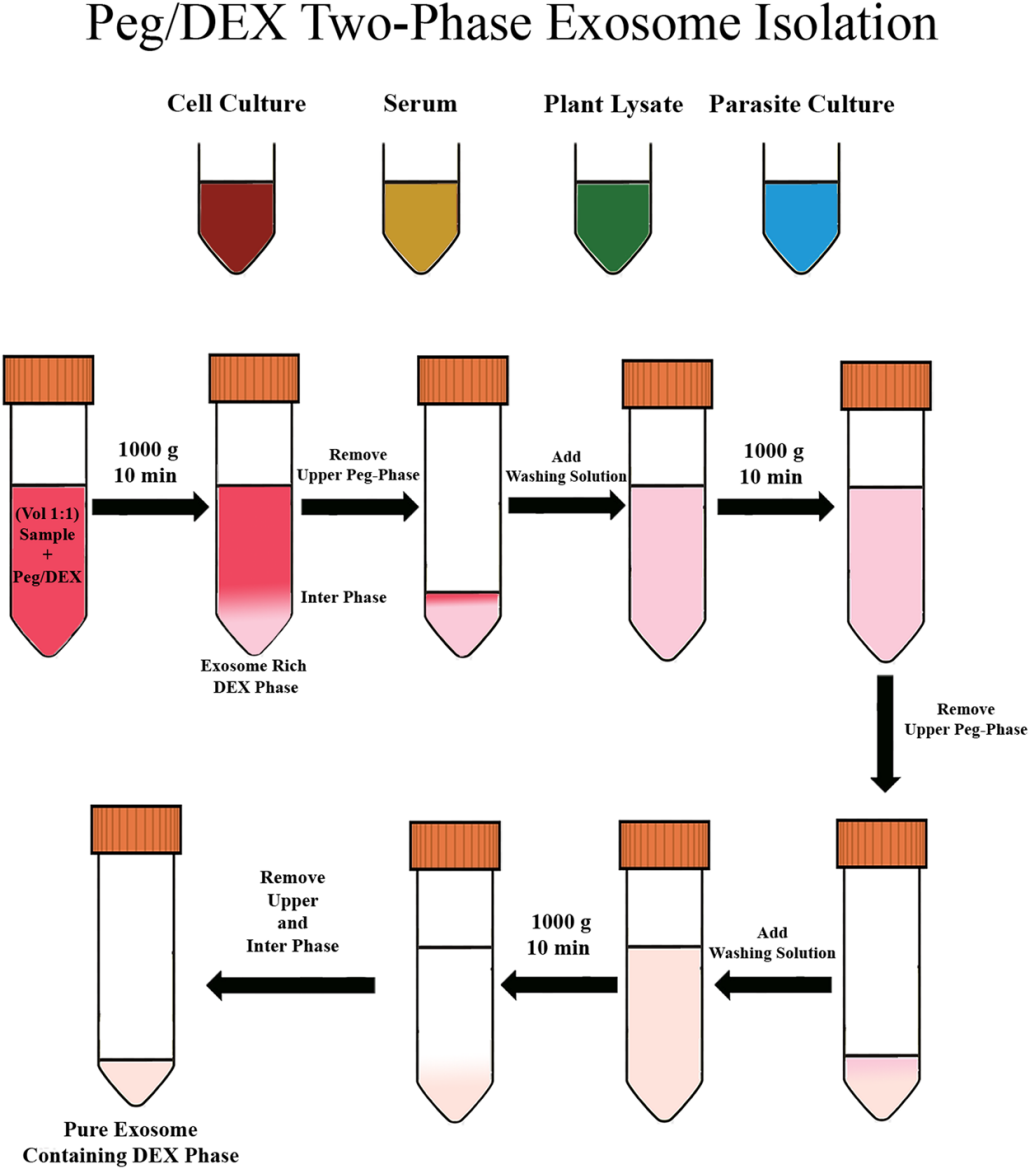
Steps of the ATPS-based EV isolation protocol.

EV were isolated with a modified protocol of one described by Shin *et al*.^20^. Isolation procedure was briefly abstracted in Fig. 6.

Plants (*Punica granatum*) were obtained from a local market, were washed and juiced whole in a clean blender. Resulting lysate was passed through a cheesecloth to remove larger contaminants.

20 ml of plant lysate, cell culture media, blood serum, or parasite culture media were then centrifuged at 10,000 g for 10 minutes to remove larger contaminants. Supernatants were then passed through 0.22 *μ*m filters to remove smaller contaminating particles, and to reduce the concentrations of larger extracellular vesicles. Filtered supernatants were then mixed at a 1:1 volume ratio with ATPS-EV isolation solution, and were then centrifuged at 1,000 g for 10 minutes for phase separation. Twice, 80% of the volume was removed from the upper, PEG-rich phase replaced with the upper phase of washing solution, prepared by mixing ATPS-EV isolation solution with distilled water at a 1:1 volume ratio and centrifuging at 1,000 g for 10 minutes, as described in Kim *et al*.^21^.

### Characterization of the aqueous two-phase system

Construction of the binodal curve for PEG35/Dextran500 system, calculation of phase compositions and the characterization of the PEG/Dextran ATPS system used in this study was done through the volume and density measurements of the equilibrated phases as previously described^22^. Density of the phases were measured with a density meter (Anton Paar, AMA4100M), accurate to 0.0001 g/cm^3^. Volume of the phases was determined by visually locating the phase-separation line between the phases, and extracting the bottom phase with the use of a micropipette. To visualize the interfacial layer of ATPS, Coomassie Brilliant Blue R-250 was added to colourize the PEG phase, and the layer between the phases was visually confirmed.

### Nanoparticle tracking analysis

EV quantification and determination of EV size distribution were done using Nanosight NS300 (Malvern Instruments) with a 488 nm laser. Samples were diluted to the suggested concentration range of the device, showing between 20–200 in frame. Video capture was done at camera level 16 for 60 second intervals. At the end of each capture, sample was introduced to the flow cell to flush the previous portion of the sample. A total of five captures were taken for each sample. Samples were analyzed with appropriate threshold settings.Video capture and analysis was done using NTA software version 3.4.

### EV quantification using BCA

EV concentrations were evaluated by using a Pierce BCA Protein Assay Kit (Thermo Fisher) according to the manufacturer’s instructions. Bovine Serum Albumin (BSA) was used for the standard. Results were measured with a plate reader (BioTek Instruments, Inc., VT, USA).

### Characterization of EV surface antigens with flow cytometry

EVs surface markers were analyzed using Flow Cytometry. 5 *μ*g of EVs were adhered onto 5 *μ*L of (4% w/v, 4 *μ*m) aldehyde/sulfate latex beads (ThermoFisher, A37304), and incubated for 15 minutes on a shaker at RT. Next, 200 *μ*L of 2% BSA in PBS solution was added to the EV-bead mixture for 2 hours on a shaker to prevent non-specific antibody binding. Next, enough glycine solution (Merck) was added to the solution to reach a concentration of 100 mM. EV samples were incubated for 30 minutes with glycine on a shaker. After incubation, 800 *μ*L of cold PBS was added to the samples, and samples were centrifuged at 2,700 × g for 3 min. Resulting EV pellet was resuspended in 500 *μ*L of PBS, and aliquoted into 5 tubes of 100 *μ*L to be incubated with different antibodies. Conjugated antibodies of common EV markers, CD9 (Biolegend, 124808) CD63 (Biolegend 143904), CD81(Biolegend, 349506) and HSP70 (Biolegend 648004) were added at 1:1,000 dilutions to each sample, and incubated overnight. Primary antibodies of Alix (Abcam,ab186429), TSG101 (Abcam, ab209927), CALX (Abcam, ab203439) were incubated overnight, centrifuged at 2,700 × g for 3 minutes to wash the samples of excess antibodies and then resuspended in 100 *μ*L of 1:100 dilution Alexa Fluor 488 (Abcam, ab150077). Flow cytometric analysis of EVs were conducted using a Becton Dickinson (BD) FACSCalibur Flow Cytometry System (Becton Dickinson, San Jose, CA, USA).

### Western blotting

Antibodies for EV markers of CD9 (CellSignalling, 13174, 1:250), Flotillin (CellSignalling, 18634, 1:1000) and GM130 (CellSignalling, 12480, 1:1000) were tested using Western Blotting, using anti-rabbit secondary antibody (CellSignalling, 7074, 1:2000). EVs were separated by 4–20% Gradient Sodium Dodecyl Sulphate-Polyacrylamide Gel (Biorad, 4–20% Mini-PROTEANÂ® TGX^TM^, USA) Gel and Electrophoresis (SDS-PAGE) were applied. Proteins were transferred onto PVDF membrane (Bio-Rad #162-0177, USA) by Pierce G2 fast blotter. Membranes were blocked with 5% skim milk powder, for 1 hour and 30 minutes at room temperature. After the overnight incubation with primary antibody at 4 °C. Membrane was visualized with ChemidocTM XRS + system. Image Lab Software 6.0.1 was used for quantification of images.

### Scanning electron microscopy

EV samples were diluted 1:100 with distilled water and dried on carbon adhesive disks for imaging. SEM imaging was performed in a FEI ESEM QUANTA 250 FEG environmental scanning electron microscope without sputter coating. Samples were imaged at low vacuum mode using the low vacuum secondary electron detector LFD (large field detector) under a pressure of 100 Pascal and at an accelerating voltage of 20 kV.

### Identification of fatty acid methyl esters (FAME)

Fatty acid profiles of EVs derived from cell culture, serum, parasite culture, and EV-like nanoparticles from plant lysates were determined by isolating fatty acids and transforming them into Fatty Acid Methyl Esters (FAME). Sample preparation and analysis with Agilent Tech GC-Midi 6890 N were performed following the manufacturer’s instructions. 1 mL of Reagent 1 consisting of 15% NaOH, 50% Methanol was added to each tube containing EV samples. Then, the tubes were vortexed for 5–10 times and heated in a boiling water bath for 5 min. Afterward, the tubes were vortexed again and returned to the water bath incubation for 25 min heating. After the completion of incubation, the tubes were cooled in a water bath at 4^o^C for ca. 1 min. 2 mL of Reagent 2 consisting of 10% HCl, 45% Methanol was added to the samples for methylation reaction. The tubes were vortexed for 5–10 times and heated in a water bath at 80 °C for 10 min and cooled again. Then, 1,25 mL of Reagent 3 consisting of 50% Hexane, 50% Methylester-butyl ether was added to the samples for extraction of fatty acids. After centrifugation at 3,000 rpm for 10 minutes, two phases were formed. The upper phase was transferred to a clean tube and the lower phase was discarded. 3 mL of reagent 4 consisting of 10% NaOH was added to the upper phase and centrifuged at 3,000 rpm for 5 min. Then, the upper phase was transferred to GC vial and insert for GC-MS analysis. Searching results for fatty acids were confirmed manually and by searching The Sherlock database using the The Sherlock^TM^ Microbial Identification System (MIS).

### Statistical analysis

All experimental data in this study were analyzed using Graphpad Prism7 software. One-way ANOVA was used to evaluate the statistical significance of the differences among the experimental groups. For results with p values that are less than 0.05, which is accepted as alpha value, the difference among the groups was deemed significant. Each experiment was repeated thrice.

## Supplementary information

Supplementary Information

## Data Availability

Datasets used and/or analysed during the current study are available from the corresponding author on reasonable request.
