# Supplementary material for: Optimized Isolation of Extracellular Vesicles From Various Organic Sources Using Aqueous Two-Phase System
Source: Sci Rep. 2019 Dec 16;9:19159. doi: 10.1038/s41598-019-55477-0 (PMC6915764; doi:10.1038/s41598-019-55477-0)
Supplement: Supplementary file 1 — Supplementary Information [file 41598_2019_55477_MOESM1_ESM.pdf]

Optimized Isolation of Extracellular Vesicles From Various Organic Sources Using Aqueous Two Phase System

Oğuz Kaan Kırbaş[1], Batuhan Turhan Bozkurt[1], Ayla Burçin Asutay[1], Beyza Mat[1], Bihter Özdemir[1], Dilek Öztürkoğlu[1], Hülya Ölmez[2], Zeynep İşlek[1], Fikrettin Şahin[1], and Pakize Neslihan Taşlı[1*]

[1]Yeditepe University, Faculty of Engineering and Architecture, Department of Genetics and Bioengineering, Kayisdagi St., 34755, Istanbul, Turkey.
[2]Tubitak Marmara Research Center, Baris Dist,41470, Kocaeli, Turkey.
[*]pneslihan.tasli@yeditepe.edu.tr

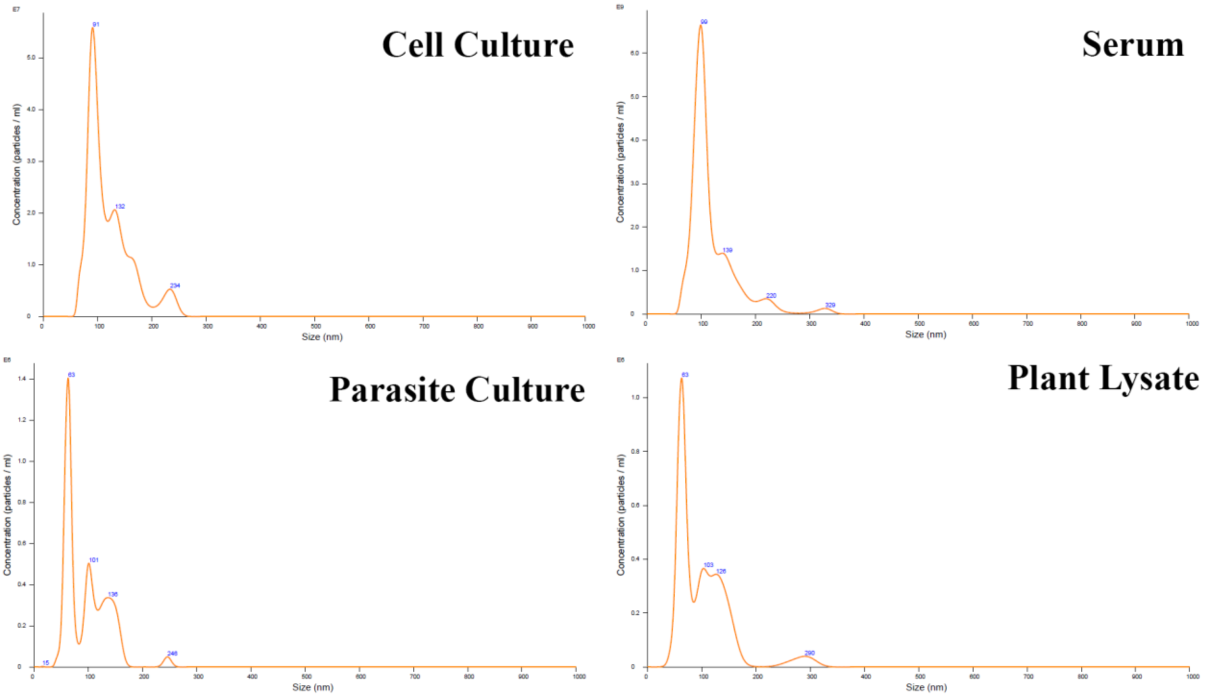

Supplemantary figure 1: NTA (Nanoparticle Tracking Analysis) data shows concentration (particles/mL) and size (nm) distrubution of EVs isolated from Cell Culture, Serum, Parasite Culture and Plant Lysate samples.

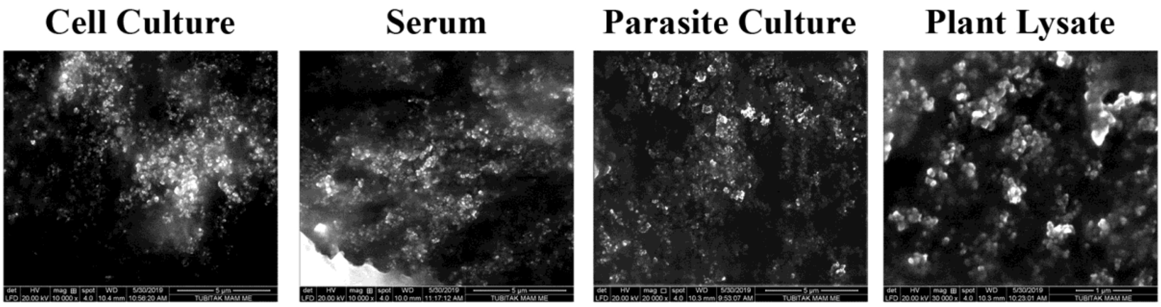

Supplemantary figure 2: Wide range SEM images of EVs isolated from Cell Culture, Serum, Parasite Culture and Plant Lysate samples.

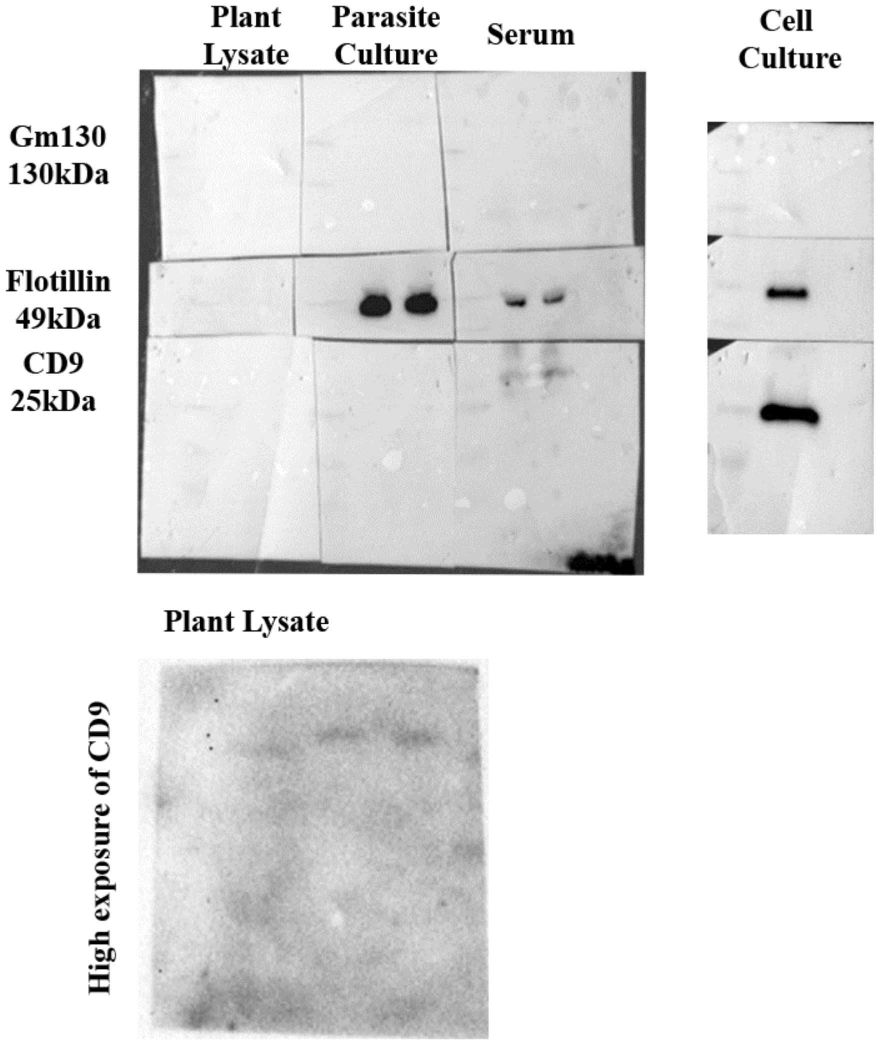

Supplemantary figure 3: Complete gel images for Western Blot Analysis of EVs isolated from Cell Culture, Serum, Parasite Culture and Plant Lysate samples.
